# Relationships of the arcuate fasciculus and nigrostriatal tract with language ability in intracerebral hemorrhage using a diffusion tensor imaging

**DOI:** 10.1038/s41598-023-36307-w

**Published:** 2023-06-06

**Authors:** Sung Ho Jang, Sang Seok Yeo, Min Jye Cho

**Affiliations:** 1 Department of Physical Medicine and Rehabilitation, College of Medicine, Yeungnam University, 317-1, Daemyung dong, Namgu, Daegu, 705-717 Republic of Korea; 2 Department of Physical Therapy, College of Health Sciences, Dankook University, 119, Dandae-ro, Dongnam-gu, Cheonan-si, Chungnam 330-714 Republic of Korea

**Keywords:** White matter injury, Stroke

## Abstract

We investigated the relationships of the arcuate fasciculus (AF) and the nigrostriatal tract (NST) with the language ability in patients with putaminal hemorrhage (PH) in the dominant hemisphere, using diffusion tensor tractography (DTT). Twenty-seven consecutive right-handed patients with PH and 27 age- and sex-matched normal control subjects were recruited. The aphasia quotient (AQ) score was used to evaluate the language ability at the early stage (within six weeks after onset). The fractional anisotropy (FA) value and tract volume (TV) of the ipsilesional AF and the ipsilesional NST were measured. The FA values and TVs of the ipsilesional AF and the ipsilesional NST of the patient group were lower than those of the control group (*p* < 0.05). The AQ score showed no significant correlation with the FA values of the ipsilesional AF and the ipsilesional NST (*p* > 0.05). By contrast, the AQ score showed a strong positive correlation with the TV of the ipsilesional AF (*r* = 0.868, *p* < 0.05). In addition, the AQ score revealed a moderate positive correlation with the TV of the ipsilesional NST (*r* = 0.577, *p* < 0.05). The states of the ipsilesional AF and the ipsilesional NST were closely related to the language ability at the early stages in patients with PH in the dominant hemisphere. Furthermore, the ipsilesional AF was more closely related to the language ability than the ipsilesional NST.

## Introduction

Aphasia is a severe disabling sequela of stroke; approximately 24–38% of acute stroke patients suffer from aphasia^1–5^. Several neural tracts, including the arcuate fasciculus (AF), are involved in the language ability of the human brain^6–12^. Elucidation of the neural correlates for aphasia in stroke patients is clinically important because it may help clinicians set rehabilitation strategies and predict the outcome of aphasia.

Among the neural tracts for language ability, the AF in the dominant hemisphere has been considered the most representative neural tract in the human brain^10,13^. The AF, which connects the caudal temporal cortex (Wernicke’s area) and inferior frontal lobe (Broca’s area), is an essential neural tract for language processing^14–18^. An injury to the AF could cause variable language problems, such as conduction aphasia, progressive aphasia, tone-deafness, dyslexia, and stuttering^18–21^. Many studies have reported the relationship of the AF with aphasia in stroke patients^6–12^. By contrast, relatively little is known about the other neural tracts^22,23^.

The nigrostriatal tract (NST), which is one of the major dopaminergic neural pathways that originate from the substantia nigra pars compacta in the midbrain and terminate to the dorsal striatum (caudate nucleus and putamen), plays an important role in the neurochemical foundations of speech by assigning dopamine transmission from the ventral portion of the substantia nigra pars compacta^24–26^. In addition, the NST has been reported to regulate the activity of the laryngeal motor cortex and the vocal basal ganglia circuitry during speech production^24–26^. As a result, the administration of dopaminergic agonists, such as levodopa, a dopamine D2 receptor agonist, has been considered useful for the recovery of post-stroke aphasia^26,27^.

A few studies reported that language disability was associated with nigrostriatal degeneration in some brain pathologies, such as non-fluent/agrammatic variant of primary progressive aphasia and Huntington’s disease, using Single Photon Emission Computed Tomography (SPECT) and voxel-based morphometry^28,29^. On the other hand, the relationship of the NST with aphasia in stroke patients has not been reported. Recently, diffusion tensor tractography (DTT) was used as a three-dimensional reconstruction method for the NST in the human brain^30^. This study hypothesized that the NST and AF are related to the language ability in stroke patients.

In this study, we investigate the relationships of the AF and the NST with the language ability in patients with putaminal hemorrhage (PH) in the dominant hemisphere, using DTT.

## Methods

### Subjects

Twenty-seven consecutive right-handed patients with PH (17 men and 10 women; mean age 49.78 ± 10.03 years; range, 28–67 years) and 27 age- and sex-matched normal control subjects (17 men and 10 women; mean age 43.48 ± 10.09 years; range, 26–63 years) were recruited for this study. All patients were recruited according to the following inclusion criteria: (1) first-ever stroke; (2) aged 20–69 years; (3) spontaneous PH on the left hemisphere confirmed by a neuroradiologist; (4) hemiparesis contralateral to PH lesion; (5) diffusion tensor imaging (DTI) scanning and language function evaluation were performed within six weeks after onset; (6) no previous history of psychiatric and neurological disease. Table 1 lists the demographic and clinical characteristics of the patient and control groups. This study was performed retrospectively and conducted in accordance with the guideline of the Declaration of Helsinki. All of the subjects provided signed, informed consent and the study protocol was approved by the institutional review board of the Yeungnam University Hospital (IRB number: YUMC 2021-03-014).

**Table 1:** Demographic and clinical data of the patient and control groups.

	Patient	Control
Age (years)	49.78 ± 10.03	43.48 ± 10.09
Number (n)	27	27
Male:female	17:10	17:10
Mean duration to AQ evaluation (days)	18.26 ± 9.49	–
AQ score	55.44 ± 33.27	–

*AQ* aphasia quotient; Values presented are means ± standard deviations.

### Language function evaluation

The aphasia quotient (AQ) of the Western Aphasia Battery was used to evaluate the language ability of the subjects (range: 0–100 percentiles). The AQ score consisted of four subset scores (fluency, comprehension, repetition, and naming), with higher scores indicating better functioning^31^. The reliability and validity of the Western Aphasia Battery have been well established^31,32^. The AQ score was acquired at an average of 18.26 ± 9.49 days after PH onset.

### Diffusion tensor imaging and tractography

**Figure 1:**
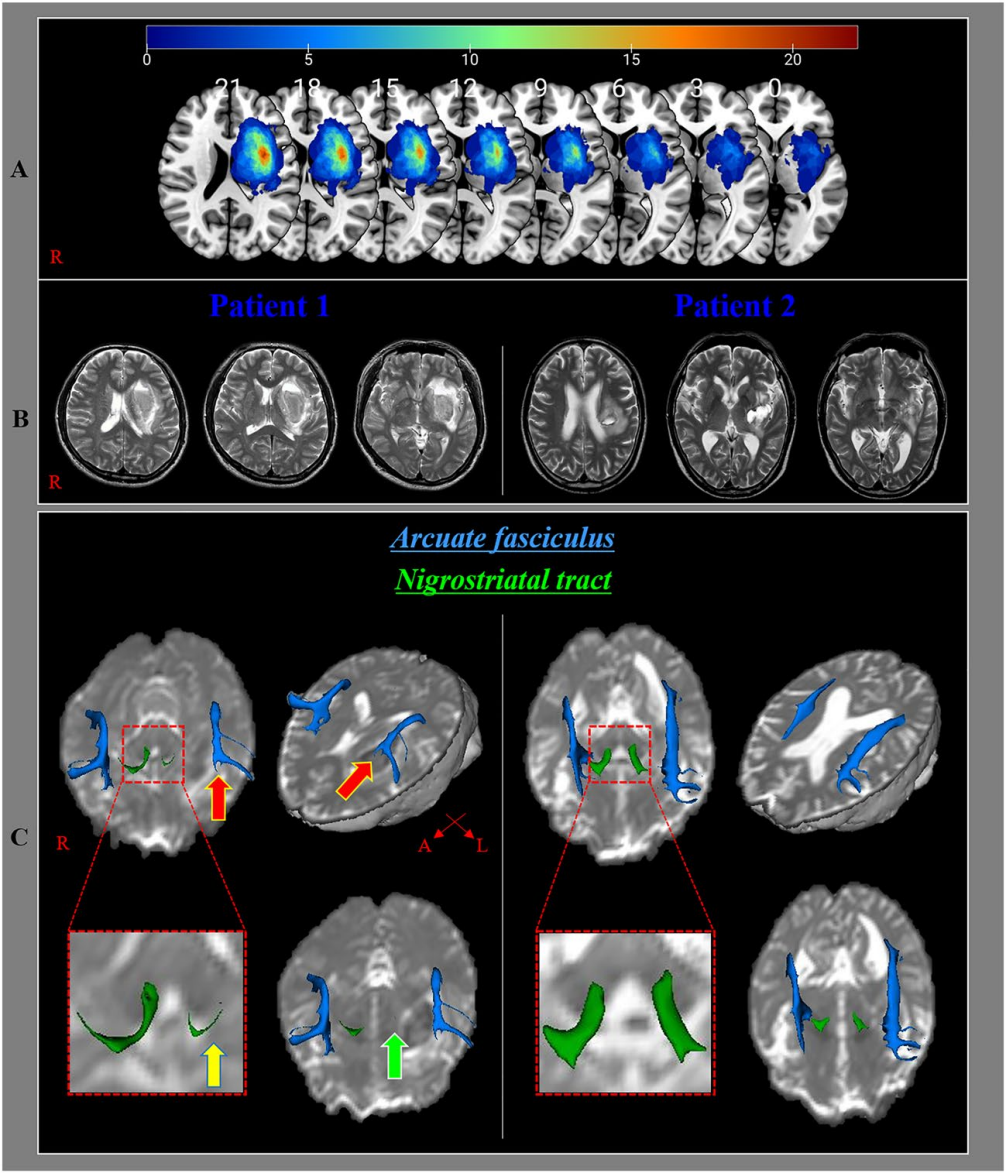
Results of diffusion tensor tractography for the arcuate fasciculus (AF) and the nigrostriatal tract (NST). (**A**) The lesion maps of all 27 patients are overlaid on an MNI152 standard-space T1-weighted template. MNI z coordinates of each transverse section are provided. Most lesioned voxels are located in the lentiform (putamen and globus pallidus) and the corona radiata region. (**B**) T2-weighted brain magnetic resonance images at the time of diffusion tensor imaging scanning in representative patients with a putaminal hemorrhage at the early stage [patient 1:55-year-old male with aphasia quotient (AQ) score 33.1, patient 2: 63-year-old male with AQ score 100]. (**C**) The ipsilesional AF (red arrow) and the ipsilesional NST (yellow arrow) at the early stage in patient 1 have a lower tract volume than those in patient 2. In addition, the ipsilesional NST (green arrow) at the early stage in patient 1 shows discontinuation at the lesion, whereas the integrity of the ipsilesional NST at the early stage in patient 2 was preserved.

The DTI data of patients were obtained at a similar time (within two days before and after DTI scanning) with a language (AQ) evaluation (18.26 ± 9.49 days after PH onset). DTI was performed using a sensitivity-encoding head coil on a 1.5 T Philips Gyroscan Intera (Hoffman-LaRoche Ltd, Best, Netherlands) scanner with single-shot echo-planar imaging and navigator echo. Sixty-seven contiguous slices (acquisition matrix = 96 × 96; reconstruction matrix = 192 × 192; field of view = 240 mm × 240 mm; TR = 10,726 ms; TE = 76 ms, b = 1000 s/mm^2^, NEX = 1, and thickness = 2.5 mm) were acquired for each of the 32 noncollinear diffusion-sensitizing gradients. Image distortion due to eddy current and head motion effects were corrected using affine multi-scale two-dimensional registration at the Oxford Centre for Functional Magnetic Resonance Imaging of Brain (FMRIB) Software Library (FSL: https://www.fmrib.ox.ac.uk/fsl) (Analysis Group, Oxford, UK)^33^. Fiber tracking was performed using the tractography routines implemented in the FMRIB Diffusion Toolbox (https://fsl.fmrib.ox.ac.uk/fsl/fslwiki/FDT) to apply a probabilistic tractography method based on the multi-fiber model^34,35^. The AF was reconstructed by assigning the seed region of interest (ROI) manually on the posterior parietal area of the superior longitudinal fascicle while placing the target ROI in the posterior temporal lobe^36,37^. The NST was tracked by locating the seed ROI manually on the substantia nigra at the midbrain on the fractional anisotropy (FA) map, and the target ROI was placed on the striatum on the FA map^38,39^. Of the 5000 samples produced in the seed voxel, the results for positive contacts were visualized at a minimum threshold of 500 and 30 for the AF and NST, respectively (Fig. 1). The FA value and tract volume (TV) of the AF and the NST were measured.

### Statistical analysis

Statistical analysis was performed using SPSS 21.0 for Windows (SPSS, Chicago, IL, USA). The Kolmogorov–Smirnov test was performed to determine the normality of the AQ score and the DTT (FA value and TV) parameters of the ipsilesional AF and the ipsilesional NST. The TV of the ipsilesional AF and the FA value and TV of the ipsilesional NST did not satisfy normality (*p* < 0.05). Based on the normality of the data, an independent *t* test and Mann–Whitney *U* test were conducted to compare the DTT parameters of the ipsilesional AF and the ipsilesional NST between patient and control groups. In addition, Pearson (r value) and Spearman (rho value) correlation analyses were used to assess the significance of the correlations between the AQ score and DTT parameters of the ipsilesional AF and the ipsilesional NST; *p* < 0.05 was considered significant. The correlation coefficient represents the strength (0.29 or less: none or very weak correlation; 0.3–0.49: weak correlation; 0.5–0.69: moderate correlation; 0.7 or more: strong correlation) and direction (positive or negative) of the relationship between two variables^40^.

## Results

Table 2 summarizes the comparison of DTT parameter values between the patient and control groups. The FA values and TVs of the ipsilesional AF and the ipsilesional NST were significantly lower in the patient group compared with the control group (*p* < 0.05).

**Table 2:** Comparison of the diffusion tensor tractography parameter values between the patient and control groups.

	Patient	Control	*t*	*z*	*p*
FA of ipsilesional AF^a^	0.36 ± 0.06	0.41 ± 0.03	− 4.175	–	0.00*
TV of ipsilesional AF^b^	962.00 ± 873.25	1345.27 ± 652.76	–	− 2.344	0.02*
FA of ipsilesional NST^b^	0.31 ± 0.14	0.44 ± 0.03	–	− 4.975	0.00*
TV of ipsilesional NST^b^	122.37 ± 132.31	323.09 ± 104.05	–	− 4.698	0.00*

*FA* fractional anisotropy, *AF* arcuate fasciculus, *TV* tract volume, *NST* nigrostriatal tract, ^a^Independent *t* test, ^b^Mann–Whitney *U* test. *Statistically significant at *p* < 0.05; Values presented are means ± standard deviations.

**Figure 2:**
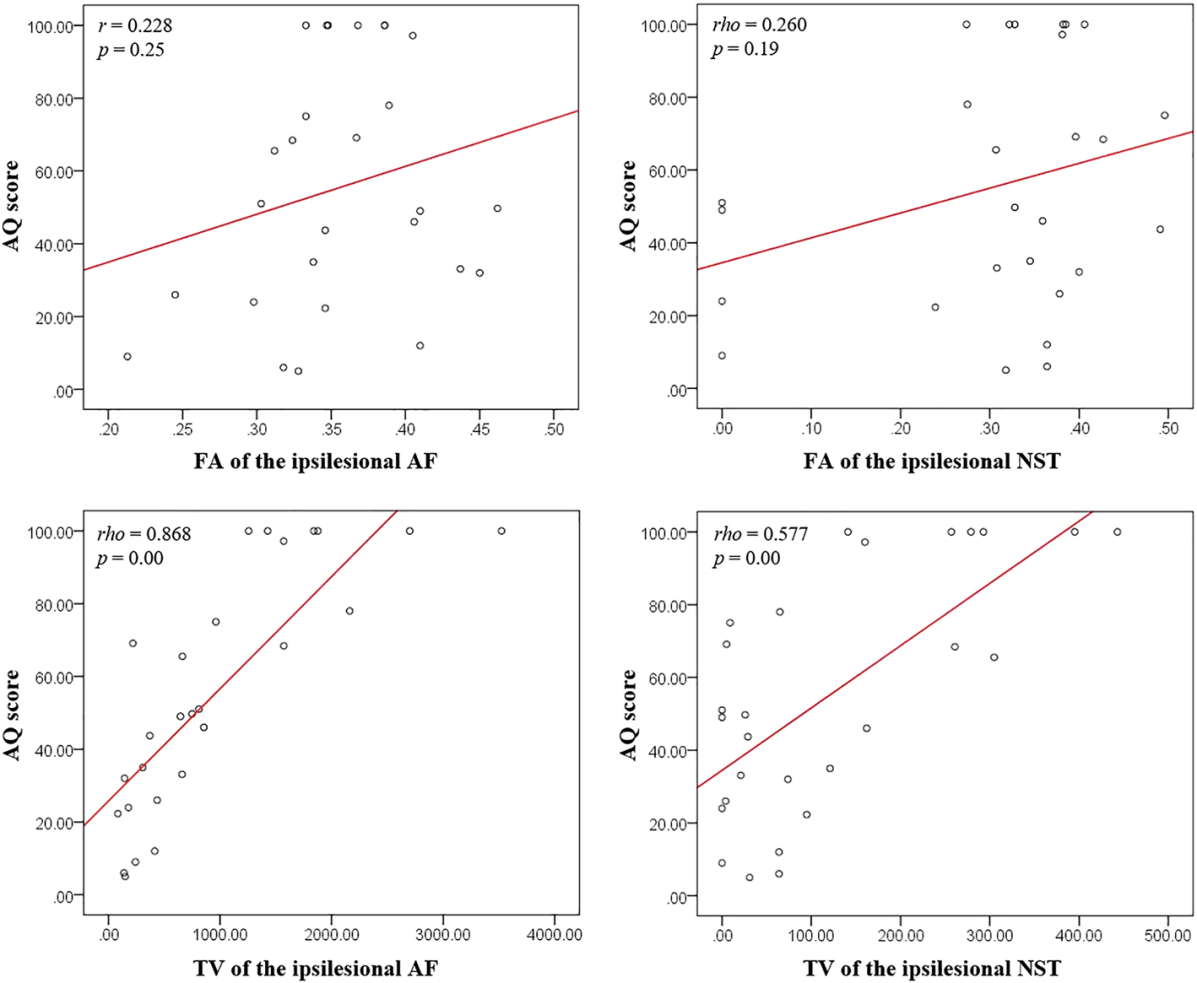
Scatter plots showing the correlation between diffusion tensor tractography parameters [fractional anisotropy (FA) and tract volume (TV)] of the ipsilesional arcuate fasciculus (AF) and the nigrostriatal tract (NST). The AQ score has a strong positive correlation with the TV of the ipsilesional AF (*r* = 0.868, *p* < 0.05), and a moderate positive correlation with the TV of the ipsilesional NST (*rho* = 0.577, *p* < 0.05).

Figure 2 represents the correlations between the AQ score and DTT parameters of the patients. The AQ score showed no significant correlation with the FA values of the ipsilesional AF and the ipsilesional NST (*p* > 0.05). On the other hand, the AQ score revealed a strong positive correlation with the TV of the ipsilesional AF (*r* = 0.868, *p* < 0.05). By contrast, the AQ score had a moderate positive correlation with the TV of the ipsilesional NST (*r* = 0.577, *p* < 0.05).

## Discussion

In this study, DTT was performed to investigate the relationships of the ipsilesional AF and the ipsilesional NST with the language ability at the early stage in patients with PH in the dominant hemisphere. These results are summarized as follows. First, FA values and TVs of the ipsilesional AF and the ipsilesional NST of the patient group were lower than those of the control group. Second, the AQ score showed a strong positive correlation with the TV of the ipsilesional AF, whereas the AQ score revealed a moderate positive correlation with the TV of the ipsilesional NST. On the other hand, the AQ score did not correlate with the FA values of the ipsilesional AF and ipsilesional NST.

The FA value indicates the degree of directionality of water molecule diffusion and the integrity of white matter microstructures, such as axons, myelin, and microtubules, showing the fiber density, axonal diameter, and white matter myelination^41,42^. The TV represents the volume of voxels within the neural tract meaning the number of neural fibers^42,43^. Therefore, our results that the low FA values and TVs of the ipsilesional AF and the ipsilesional NST in the patient group compared with those in the control group indicate decreased microstructural integrity and neural fibers in these neural structures, which suggest neural injury.

The results showed no significant correlations between the AQ score and the FA values of the ipsilesional AF and the ipsilesional NST. This suggests that the language ability of the patients was not associated with the integrity of the ipsilesional AF and the ipsilesional NST. By contrast, the significant positive correlations of the language ability with the ipsilesional AF (strong) and the ipsilesional NST (moderate) indicated that the language ability was related to the number of remaining neural fibers of the ipsilesional AF and the ipsilesional NST of the patients. Considering the correlation coefficient, the remaining neural fiber number of the ipsilesional AF was associated more closely with language ability than the ipsilesional NST.

Many studies have used DTT to demonstrate the relationship of the language disability with the AF in stroke patients^6–12^. These studies focused on the association between the language outcome and state of the AF in stroke patients with aphasia^6–12^. By contrast, a few studies reported that language disability was related to the nigrostriatal system in patients with a few brain pathologies^28,29^. In 2013, Gil-Navarro et al. demonstrated nigrostriatal degeneration in patients with a non-fluent/agrammatic variant of primary progressive aphasia through the reduced tracer uptake in the striatum using SPECT^28^. In 2018, Giavazzi et al. used voxel-based morphometry to show that in patients with Huntington’s disease, the linguistic performance was correlated with grey matter atrophy within the dorsal striatum, particularly in the caudate nucleus^29^. To the best of the authors’ knowledge, this is the first study to report the relationships between the language ability and states of the ipsilesional AF and the ipsilesional NST in patients with PH in the dominant hemisphere.

Nevertheless, this study had some limitations. First, crossing fibers or the partial volume effect during DTT analysis could produce false-positive and false-negative results^44^. Second, because this study was conducted retrospectively, the only AQ score was used to evaluate the language ability and DTI data at the early stages of PH. Third, other neural tracts related to the language ability except for the AF, such as the superior longitudinal fasciculus, were not evaluated. Fourth, this study assessed a small number of subjects. A large number of subjects could not be recruited because only right-handed patients with PH in the left hemisphere were selected. Hence, further prospective studies involving more subjects are needed.

DTT showed that the language ability was closely related to the states of the ipsilesional AF and the ipsilesional NST at the early stages in patients with PH in the dominant hemisphere. Furthermore, the ipsilesional AF was more closely related to the language ability than the ipsilesional NST. These results suggest that the states of the ipsilesional AF and ipsilesional NST at the early stages could provide useful information for establishing and maximizing various rehabilitation strategies for language ability and predicting the language outcome of patients with PH. On the other hand, further studies for various brain pathologies, including cerebral infarct and traumatic brain injury, are needed.

## Data Availability

The datasets generated during and/or analyzed during the current study are available from the corresponding author on reasonable request.
